# Cut of Clothes Maximizes the Effect of Amodal Completion to Make You Look Thinner

**DOI:** 10.1177/2041669518815705

**Published:** 2018-12-08

**Authors:** Yoshie Kiritani, Akane Kawasaki, Ikjoon Chang

**Affiliations:** Department of Design, Graduate School of Engineering, Chiba University, Japan; Department of Design Science, Graduate School of Science and Engineering, Chiba University, Japan; Department of Design, Graduate School of Engineering, Chiba University, Japan

**Keywords:** size perception, amodal completion, fashion

## Abstract

Amodal completion has various functional effects, including an apparent slimming effect achieved by clothes. Local and global completion factors have been examined in previous studies, which also apply to the apparent slimming effect. Exposed parts of the body constitute the local factor at the junction area, while the shape or cut of the clothes is concerned with the whole configuration. This study investigated which is more important, the local or whole factors, for amodal completion in relation to the apparent slimming effect using drawings as stimuli. In Experiment 1, we examined the effects of the length and cut of a skirt. The length of the skirt corresponds to the local factor of the body, that is, the legs, because the exposed parts of the legs depend on skirt length (assuming a person of consistent height). We found that skirts' cut influenced their effect more than their length did. Experiments 2 and 3 revealed that the vertical form of clothes affects slimming by hiding thicker parts of the body and highlighting thinner parts. A supplemental experiment using geometrical figures suggested that the apparent slimming effect of clothes might occur only in the human body configuration.

## Introduction

Amodal completion (Kanizsa, 1955), or the perception of a whole object that is partially occluded by other objects, takes place everywhere in our daily life. An example of the concept's possible application is seen in Morikawa (2017). This study demonstrated that drawings of the same woman can be shown to have different perceptual leg thickness (p. 256, Figure II. 26-5, upper part of the figure); a woman hiding her thigh and leg below the knee seemed to have thinner legs than one hiding her knees and her ankle region. That is, when the thin parts of the legs are visible and the thick parts are invisible, the legs look thin. Morikawa explained that this effect was an example of the illusion of amodal perceptual completion (though he used rectangles as occluders, not clothes); he says, “When only the thin parts of the legs, such as knee and ankles, are visible, the entire legs appear thin” (pp. 255–256).

According to Kanizsa (1980), what we should (or should not) see as an amodal part under the occluding object is determined by the Gestalt factor of good continuity. For example, if, in a black-and-white checkered pattern, a black square is hidden by a circle, we no longer see the black square under the circle but instead see a white cross whose central part is hidden by the circle (p. 172, Figure 4.14); in this case, the symmetry of the surrounding pattern does not affect the amodal form under the occluding circle, but the way the occluding object locally contacts for the occluded one strongly affects the form of the amodal part. Morikawa's (2017) explanation of the apparent slimming effect is the same: The width of the exposed legs connecting the rectangles determines the amodal width of the legs. In these explanations, the local T-junction is crucial.

The purpose of this study is to establish which is more important for amodal completion in relation to the apparent slimming effect by clothing: properties of the visible parts of the occluded object (the body) or of those of the occluding object (the clothes). If the local factors have a stronger effect in such a case, body parts such as legs that appear from under the clothes would determine the apparent body size under the clothes. However, some studies of amodal completion in geometrical figures indicate that to determine the amodal form, it is not enough just to consider the form of the connection of the exposed parts of the occluded object (e.g., Takashima, Fujii, & Shiina, 2009). Gyoba (2002) suggested that global factors play a more important role in amodal completion than in modal completion, such as in the Rosenbach effect, in which a perceptual transparency emerges under a piece of opaque black paper (Rosenbach, 1902). If the entire figure is more important than the local factors, the shape of the clothes would affect the apparent body size more.

**Figure 1.**
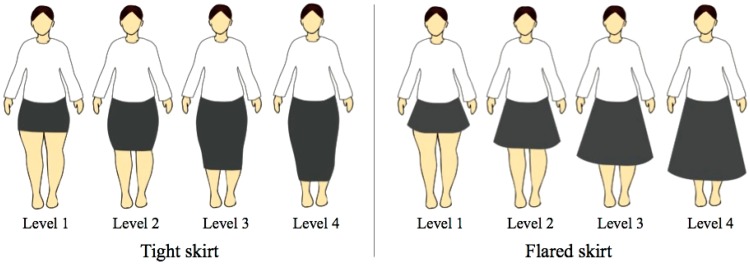
Eight standard stimuli used in Experiment 1.

This study consists of three main experiments and one supplemental experiment. Experiment 1 examined the effect of the visible parts of the legs by varying the length of a skirt and the effect of its form. The length of the skirt changes the local factor of the legs because the parts of the legs exposed depend on the length of the skirt (see Figure 1). In contrast, the form of the skirt reflects the factor of the occluding object, which is not local but corresponds to the inclusive global process. In Experiment 2, we examined the effects of the entire figure, including both vertical and horizontal forms, using four different shapes of skirt. In Experiment 3, we confirmed again the effects of vertical and horizontal forms of clothes by examining total body shapes wearing a one-piece. All stimuli used in this study were drawings. A supplemental experiment examined the effects found in the main experiments using geometrical figures. The psychophysical method of adjustment was adopted.

## Experiment 1

### Method

#### Participants

Twenty-three female graduate or undergraduate students (*M*_age_ = 20.8 years, standard deviation [*SD*] = 1.53) participated; all had normal vision. Verbal informed consent was obtained from each participant before they began the study.

#### Apparatus and stimuli

All stimuli were presented on the screen of a 13-in. MacBook Pro, 2012 version, using PsychoPy software, v.1.851 (Peirce, 2007).

**Figure 2.**
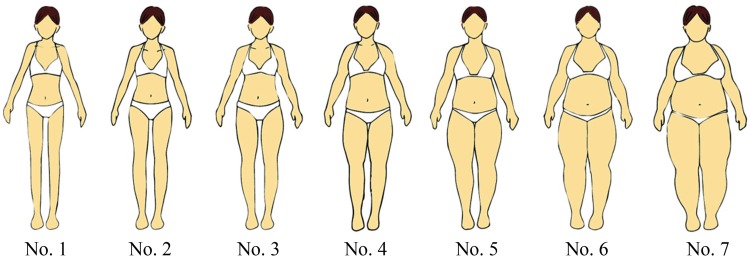
Seven comparative stimuli used in Experiments 1, 2, and 3.

Two factors, the length of the skirt and its shape, yielded eight standard stimuli (Figure 1). In all standard stimuli, the body of the woman partially occluded by the clothes was identical. This woman's upper body was that of comparative stimulus No. 4 presented below, and the lower body was that of comparative stimulus No. 5. We used a chimera body to prevent the participants from inferring the shape of the lower part of the body based on the upper body. We carefully tightly fitted these clothes to the women so that their bodies would not protrude from the clothes. There were seven comparative stimuli drawings of women in their bra and underpants (Figure 2); No. 1 was thinnest, No. 4 was the middle, and No. 7 was fattest.

Each drawing was fitted within a rectangle with a width of about 80 mm (9.15°) and a height of about 200 mm (22.62°). They were viewed from about 50 cm away.

#### Procedure

The following method of adjustment was adopted. After 500 milliseconds presentation of a fixation point in the center of the monitor while pressing the space key, one of the eight standard stimuli and a comparative stimulus were presented side by side. The participants could move through the sequence of comparative stimuli by pressing the space key, moving up and down the range to determine the best match. Ascending and descending order of first presentation of comparative stimuli and side position (left or right) of the standard stimulus and of the comparative stimulus were counterbalanced among the participants. The presentation order of the standard stimuli was randomized.

The participants selected the comparative stimulus that was perceived to have the same body size as the corresponding standard stimulus by pressing the space key. They were instructed that the judgment was about the whole body not just the lower part. Each participant made judgments of eight standard stimuli, 4 times for each stimulus. Thus, the total number of trials for each participant was 32. The experiment was done in a dark room; only the light of the monitor was a source of illumination.

### Results and Discussion

**Figure 3.**
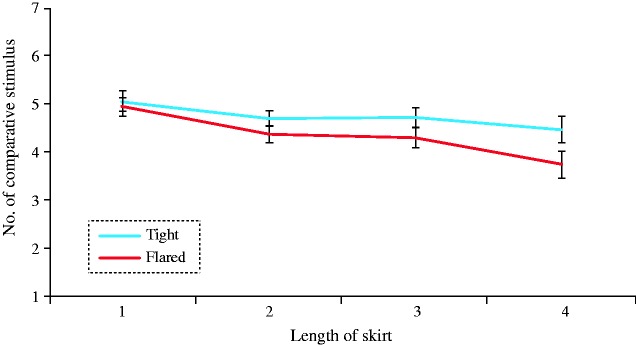
Results of Experiment 1 (error bars represent 95% Cousineau–Morey–Baguley difference-adjusted normalized confidence intervals).

Figure 3 shows the average of Point of Subjective Equality (PSE) scores. The vertical axis represents the number of the comparative stimulus. Because the standard stimulus had the lower body of comparative stimulus No. 5, the Number 5 in the vertical axis should mean that the standard stimulus appeared to be the same size as the actually illustrated body.

After adjusting the degrees of freedom using Greenhouse and Geisser's epsilon, a two-way repeated-measures analysis of variance (ANOVA) was conducted. The main effects of length of skirt, *F*(1, 22) = 53.100, *p* = .0000, $\eta_{\text{G}}^{2}$ = .365, shape of skirt, *F*(1, 22) = 33.062, *p* = .0000, $\eta_{\text{G}}^{2}$ = .191, and their interaction, *F*(1, 22) = 7.658, *p* = .0112, $\eta_{\text{G}}^{2}$ = .063, were all significant. The simple main effect of length of skirt was significant for both shapes of skirt, for the tight skirt, *F*(1, 22) = 13.527, *p* = .0013, $\eta_{\text{G}}^{2}$ = .230, and for the flared skirt, *F*(1, 22) = 50.312, *p* = .0000, $\eta_{\text{G}}^{2}$ = .473. In the tight skirt condition, Holm's sequentially rejective Bonferroni procedure revealed significant differences in PSE scores between Levels 1 and 2, *t*(22, 0.05) = 5.564, *p* = .0001, 1 and 3, *t*(22, 0.05) = 3.875, *p* = .0033, and 1 and 4, *t*(22, 0.05) =4.691, *p* = .0006; other pairs were not significant. On the other hand, in the flared skirt condition, significant differences were revealed in PSE scores between all pairs of length of skirt except for Levels 2 and 3, *t*(22, 0.05) = 0.924, *p* = .3654, *ns*.

These results show that (a) the body seemed thinner as the skirt became longer, (b) the flared skirt made the body seem thinner than the tight skirt, and (c) with a longer skirt this greater apparent slimming effect by the flared skirt was magnified further. Thus, not only the length of skirt but also its shape can affect the apparent size of the body.

The most important result is the significant interaction between the length of the skirt and its shape. Although, as expected from Morikawa (2017), the length of skirt significantly affected the apparent slimming effect, the reality is that the effect in the tight skirt was small. In other words, length of skirt had an effect only on the flared skirt.

Thus, the shape of the occluding object, clothes, cannot be disregarded. In particular, the vertical silhouette of the clothes, suggesting the body line, seems important, as the difference in shape between the tight skirt and the flared one is the degree of suggestion of body line. However, there is another difference in shape between the tight skirt and the flared one: width of bottom. If we wish to regard the vertical shape of the skirt as a more important factor for the apparent slimming effect by amodal completion, we should rule out an effect of width of hem.

An alternative explanation would be that the apparent slimming effect is due to a size contrast effect. If the length of the skirt is long, the gray skirt area becomes bigger in the stimuli. Similarly, the flared skirt is bigger than the tight skirt. In Experiment 1, the standard stimulus with the larger dark gray area might have just made the leg part look smaller. To reject this possible interpretation, we should run the experiment again using stimuli with similar size of skirt but different figures.

A further issue is that we could not rule out an effect of the shape of the upper body on the apparent slimming effect. Figure 2 shows that the maximum effect obtained in the flared skirt with Length 4 was similar to the size of comparative stimulus No. 4. Thus, one might suspect that the participants made their matches simply by choosing the comparison stimulus that had the same upper body as the standard. We do not argue for this possibility because it cannot explain why this matching occurred only in the flared skirt with Length 4. However, to avoid this possibility and to simplify the interpretation of the results, in the next experiment, we did not use the chimera body as the standard stimulus.

In the next experiment, we controlled the shape of the skirt, specifically the line and the bottom, and also controlled its area. To control the vertical appearance of the hip and leg line, we again used the tight skirt versus the flared one; to change the horizontal width of the bottom, two new skirts were also added, as described in the next section.

## Experiment 2

### Method

Except for the participants and the standard stimuli, all the stimuli, the apparatus, and the procedure were the same as in Experiment 1.

The participants were 19 female undergraduate or graduate students (*M*_age_ = 20.9 years, *SD* = 1.94) who had normal vision; 8 of them had also participated in Experiment 1. Verbal informed consent was obtained from each participant before they began the study. Three of the participants had missing values, and so we deleted these persons' data from the analysis.

**Figure 4.**
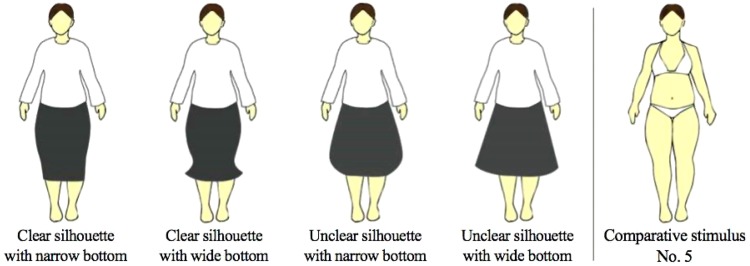
Five standard stimuli used in Experiment 2.

There were two factors used to construct the standard stimuli: the vertical silhouette of the skirt and the horizontal spread of the skirt bottom (Figure 4). The former factor corresponded to the suggestion of shape of hip and legs and was of two kinds: clear silhouette or unclear. The latter factor had two types: spreading (wider bottom) and shrinking (narrower bottom). The clear silhouette with narrow bottom skirt and the clear silhouette with wide bottom one had similar areas, as did the unclear silhouette with narrow bottom skirt and the unclear silhouette with wide bottom one. To maintain the naturalness of the skirts in the images, the wide bottom of the clear silhouette was made narrower than that of the unclear silhouette with wide bottom skirt; the length of the skirt was Length 3 in Experiment 1.

Moreover, to set a baseline, we added another standard stimulus, a drawing of a woman in her underwear. This was the same as the comparative stimulus No. 5.

### Results and Discussion

**Figure 5.**
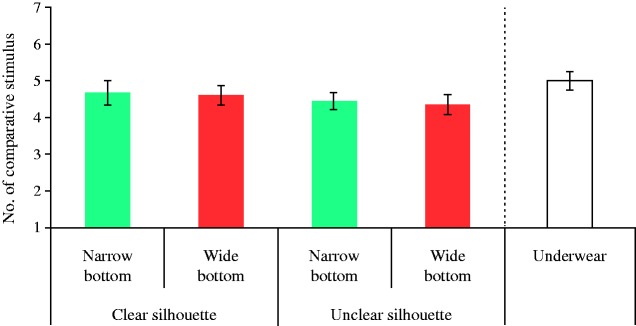
Results of Experiment 2 (error bars represent 95% Cousineau–Morey–Baguley difference-adjusted normalized confidence intervals).

Figure 5 shows the average of PSE scores. We calculated the difference between the PSEs of the standard stimuli with clothes and that of the woman in her underwear and conducted a two-way repeated-measures ANOVA. The main effect of the vertical silhouette of the skirt was marginal, *F*(1, 15) = 4.419, *p* = .0529, $\eta_{\text{G}}^{2}$ = .058; the unclear silhouette skirts might make the body seem slimmer than the clear silhouette skirts. As the length of skirt used in the stimuli of Experiment 2 was not the most effective level in Experiment 1, the vertical silhouette might not work effectively. On the other hand, the main effect of the spread of skirt bottom was clearly not significant, *F*(1, 15) = 0.974, *p* = .3393, $\eta_{\text{G}}^{2}$ = .006; the clear silhouette with wide skirt did not make the body significantly slimmer. The difference in bottom shape, as horizontal form of skirt, did not affect the apparent slimming effect. However, the results do not rule out the possibility of a size contrast effect, as the bigger skirts tended to make the body appear slimmer than the smaller skirts did.

In Experiment 3, we again confirmed the importance of vertical silhouette and tried to eliminate the possibility of a size contrast effect in examining whole-body clothes. Moreover, male as well as female participants took part in the third experiment to generalize and verify the results.

## Experiment 3

### Method

All stimuli, apparatus, and procedure were the same as in Experiment 1, except for the participants and the standard stimuli.

The participants were 21 male students (*M*_age_ = 21.9 years, *SD* = 1.75) and 23 female students (*M*_age_ = 20.8 years, *SD* = 1.61) at undergraduate and graduate levels, who had normal vision; 19 of them had also participated in Experiment 1 or 2. One female participant and two male participants had missing data, and so their data were excluded from the analysis. Verbal informed consent was obtained from each participant before they began the study.

**Figure 6.**
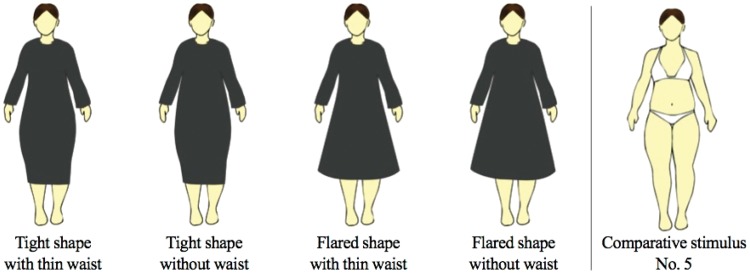
Five standard stimuli used in Experiment 3.

There were two factors for the standard stimuli: shape of skirt and shape of waist; there were two types of skirt shape, tight and flared, while the waist factor had two levels, thin waist and no waist (Figure 6). This waist factor was regarded as representing vertical shape of skirt. The drawing in underwear identical to comparative stimulus No. 5 was again used as a standard stimulus.

### Results and Discussion

**Figure 7.**
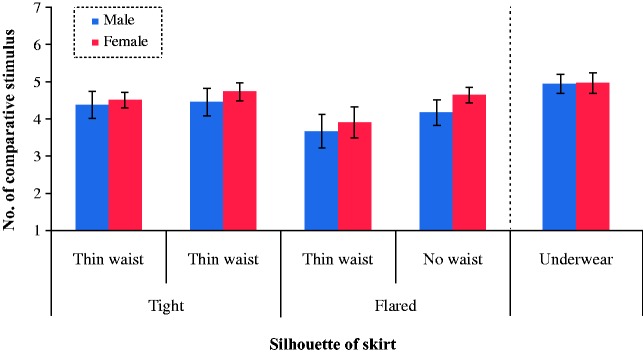
Results of Experiment 3 (error bars represent 95% Cousineau–Morey–Baguley difference-adjusted normalized confidence intervals).

Figure 7 shows the average of PSE scores. As in Experiment 2, difference scores were calculated and analyzed by a three-way mixed-design repeated-measures ANOVA. Main effects of shape of skirt, *F*(1, 39) = 22.547, *p* = .0000, $\eta_{\text{G}}^{2}$ = .105, and shape of waist, *F*(1, 39) = 24.091, *p* = .0000, $\eta_{\text{G}}^{2}$ = .087, were significant, indicating that the flared one-piece significantly made the body look slimmer than the tight one. The one-piece with thin waist had a significantly greater perceptual slimming effect than the one-piece without waist; the interaction between these two factors was also significant, *F*(1, 39) = 12.086, *p* = .0013, $\eta_{\text{G}}^{2}$ = .034. A simple main effect of the shape of skirt in the one-piece with thin waist condition was significant, *F*(1, 39) = 24.667, *p* = .0000, $\eta_{\text{G}}^{2}$ = .203; a simple main effect of the same factor in the one-piece without waist was also significant, but the effect size was small, *F*(1, 39) = 4.985, *p* = .0314, $\eta_{\text{G}}^{2}$ = .026. Finally, a simple main effect of the shape of the waist in the flared skirt was significant, *F*(1, 39) = 28.010, *p* = .0000, $\eta_{\text{G}}^{2}$ = .167.

As in Experiment 1, the flared type apparently made the body look slimmer than the tight one; and as expected based on Experiment 2, the thin waist changing a vertical silhouette was also effective. The importance of the overall state of the body was confirmed in Experiment 3. As there were no significant main effects of gender, *F*(1, 39) = 3.069, *p* = .0877, $\eta_{\text{G}}^{2}$ = .040, and no interactions with other factors, with silhouette of skirt, *F*(1, 39) = 0.666, *p* = .4913, $\eta_{\text{G}}^{2}$ = .004 and with shape of waist, *F*(1, 39) = 1.387, *p* = .2461, $\eta_{\text{G}}^{2}$ = .005, these effects of the shapes of clothes can be deemed universal.

The result in which the thin-waist one-piece made the body look slimmer than the one-piece without waist did means that the effect ruled out the size contrast effect. If the size contrast had been the cause, the one-piece without waist would have made the body look slimmer, while in fact the opposite results were obtained.

## Supplemental Experiment

**Figure 8.**
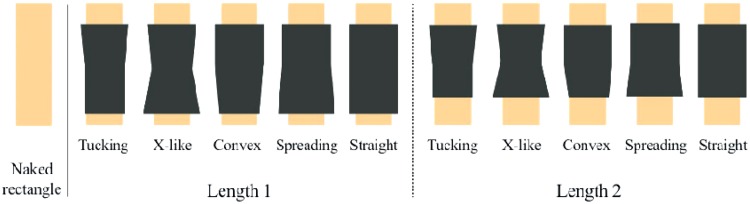
Eleven standard stimuli used in the supplemental experiment.

We confirmed the importance of the whole body's disposition for the apparent slimming effect of clothes. From the perspective of amodal completion, the shape of the occluding object, the clothes, was more crucial than the factor at the junction. At this point, we were curious about the intrinsic features of this phenomenon. In geometrical figures, distinct from drawings of women, does the effect confirmed in the former experiments appear? To investigate this, we prepared the stimuli shown in Figure 8 and measured whether the central flesh-colored rectangles under the dark gray figures seemed thinner than the *naked* rectangle.

The standard geometrical figure stimuli contained two factors: the length of the gray occluding figure and its shape. Length of the occluding figure had two levels: long (Length 2, about 155 mm, or 17.62°) and short (Length 1, about 127 mm, or 14.48°). As for the shape factor, there were five types of shape: tucking, X-like, convex, spreading, and straight. A flesh-colored rectangle without the occluding figure was also used; it was the same as the comparative stimulus No. 5, with a width of about 63.5 mm (7.27°) and a height of about 212 mm (23.94°). In all standard stimuli with the occluding figure, the height of the upper part of the flesh-colored rectangle over the occluding figure was about 35 mm (4.01°), and the width of the upper side of the occluding figure was about 85 mm (9.72°).

The seven comparative stimuli flesh-colored rectangles of gradually increasing width were prepared. The width of the narrowest rectangle was about 35 mm and that of the widest one about 78 mm (8.92°); the variation in width was about 7 mm (0.80°). The convex or the concave of the occluding figure was at 35 mm from its upper side, the width of the concave point was about 71 mm (8.12°), and the width of the convex point was about 85 mm (9.72°). The lower side of the tucking tight occluding object was about 71 mm and that of the spreading flared one was about 99 mm (11.31°).

The PSE of each standard stimulus was measured by a method of adjustment, as in the former experiments. Twenty male students (*M*_age_ = 22.0 years, *SD* = 1.90) and 21 female students (*M*_age_ = 21.2 years, *SD* = 1.60) at undergraduate or graduate level participated; 14 of the male students had also participated in Experiment 3 and 20 female students had participated in one or more of the former three experiments. Two male participants had missing data, so their data were excluded from the analysis.

**Figure 9.**
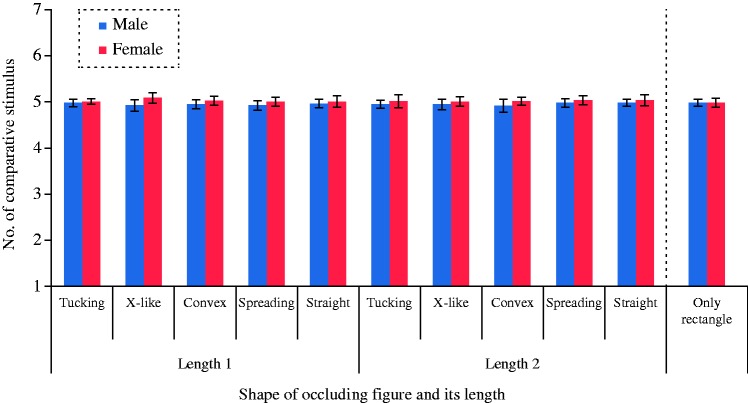
Results of the supplemental experiment (error bars represent 95% Cousineau–Morey–Baguley difference-adjusted normalized confidence intervals).

Figure 9 shows the average of PSE scores. Obviously, each stimulus got similar scores, near 5. For confirmation, the differences in scores between the stimulus with occluding figure and the stimulus without it were calculated and analyzed. A three-way mixed-design repeated-measures ANOVA revealed neither significant main effects, for gender (A), *F*(1, 37) = 2.991, *p* = .0920, $\eta_{\text{G}}^{2}$ = .033; for length of occluder (B), *F*(1, 37) = 0.003, *p* = .9584, $\eta_{\text{G}}^{2}$ = .000; and for shape of occluder (C), *F*(4, 148) = 0.349, *p* = .8443, $\eta_{\text{G}}^{2}$ = .003, nor significant interaction effects, for AB, *F*(1, 37) = 0.040, *p* = .8429, $\eta_{\text{G}}^{2}$ = .000; for AC, *F*(4, 148) = 0.859, *p* = .4903, $\eta_{\text{G}}^{2}$ = .006; for BC, *F*(4, 148) = 1.168, *p* = .3274, $\eta_{\text{G}}^{2}$ = .007; and for ABC, *F*(4, 148) = 0.673, *p* = .6116, $\eta_{\text{G}}^{2}$ = .004.

Thus, we confirmed that the apparent slimming effect did not appear in the figural pattern and that this effect may occur only with the human body configuration. In the figural patterns examined here, the width of the upper exposed part of the occluded figure was the same as the width of its lower exposed part so that the results cannot be compared simply among the experiments. We avoided the possibility that the figural stimuli would be perceived as being like a human body because we wanted to compare the results of Experiments 1, 2, and 3 to the results for amodal completion of simpler geometrical figures. Kanizsa (1980, pp. 309–321) showed that a square under a black rectangle seemed to be slimmer than a square not under the rectangle and that a wider rectangle induced this phenomenal shrinkage much more; the occluded object was a square so that the width of the upper side was the same as the width of the lower side. Although the configuration in Kanizsa was different from our stimuli, same-width up-and-down sides of the occluding figure seem not crucial to the apparent slimming effect.

Moreover, the results again deny the possibility that the size contrast effect can explain the effect confirmed in Experiment 1. In the supplemental experiment, there was no significant effect of length of the occluding figure. If the size contrast had an effect, the figures with the occluding figure with Length 2 would have made the occluded figure slimmer. However, this did not occur.

## General Discussion

The apparent slimming effect of clothes related to amodal completion was investigated; experiments revealed that the overall shape of the clothes was more important that than the local factors at the junction. For the apparent slimming effect, the local factor at the junction in amodal completion corresponds to the appearance of the legs. The approach by Morikawa (2017) to apparent slimming by amodal completion focused on the exposed part of the occluded object and concluded that to make the legs look slender the thinner part should be exposed and the thicker part should be covered. Although this is true, fairly global factors should be also considered in amodal completion. If, as Takashima et al. (2009) indicated, context or frame of reference plays an important role in amodal completion of figures, it will be insufficient to look at only the shape of the exposed part of the occluded body in the apparent slimming effect by clothes and will instead be informative, as well as natural, to focus on the whole configuration of the occluded and occluding objects. Thus, this study examined how important the shape of the occluding object—clothes—was and showed that the vertical line was more crucial.

Thus, if the slimmer part in the body is suggested and the thicker part is hidden by clothes, the whole body seems slim. Although professional stylists, who have many empirical rules, would not be surprised at these results, we nevertheless provide them here with a new theoretical underpinning. The important point is that which part(s) of the body are covered by clothes or shown, and how is a secondary concern; the more important factor is the vertical appearance of the entire body with clothes.

As for limitations of this study, we note three points. First, many female participants participated repeatedly in the experiments, which might affect the coherency of the results or bias them. However, in Experiment 3, we also included male students, who were new to the experiment, and got similar results to the female students. Thus, we regard this overlap and gender imbalance of participants as being of no matter. Second, we examined the apparent slimming effect of clothes using only one size of drawing as the standard stimulus—a size considered only a little bit fat. It is unclear whether the results of this study can be generalized using images of fatter women, for example. Third, the stimuli used were drawings, and the applicability of the results to real human bodies is still unclear. These two points should be examined in the further research.
